# Measles outbreak risk in Pakistan: exploring the potential of combining vaccination coverage and incidence data with novel data-streams to strengthen control

**DOI:** 10.1017/S0950268818001449

**Published:** 2018-06-04

**Authors:** Amy Wesolowski, Amy Winter, Andrew J. Tatem, Taimur Qureshi, Kenth Engø-Monsen, Caroline O. Buckee, Derek A. T. Cummings, C. Jessica E. Metcalf

**Affiliations:** 1 Department of Epidemiology, Johns Hopkins Bloomberg School of Public Health, Baltimore, USA; 2 Department of Ecology and Evolutionary Biology, Princeton University, Princeton, USA; 3 Department of Geography and Environment, University of Southampton, Southampton, UK; 4 Fogarty International Center, National Institutes of Health, Bethesda, USA; 5 Flowminder Foundation, Stockholm, Sweden; 6 Telenor Research, Oslo, Norway; 7 Department of Epidemiology, Harvard T.H. Chan School of Public Health, Boston, USA; 8 Center for Communicable Disease Dynamics, Harvard T.H. Chan School of Public Health, Boston, USA; 9 Department of Biology, University of Florida, Gainesville, USA; 10 Emerging Pathogens Institute, University of Florida, Gainesville, USA; 11 Woodrow Wilson School, Princeton University, Princeton, USA

**Keywords:** Measles, mobility, Pakistan, vaccination

## Abstract

Although measles incidence has reached historic lows in many parts of the world, the disease still causes substantial morbidity globally. Even where control programs have succeeded in driving measles locally extinct, unless vaccination coverage is maintained at extremely high levels, susceptible numbers may increase sufficiently to spark large outbreaks. Human mobility will drive potentially infectious contacts and interact with the landscape of susceptibility to determine the pattern of measles outbreaks. These interactions have proved difficult to characterise empirically. We explore the degree to which new sources of data combined with existing public health data can be used to evaluate the landscape of immunity and the role of spatial movement for measles introductions by retrospectively evaluating our ability to predict measles outbreaks in vaccinated populations. Using inferred spatial patterns of accumulation of susceptible individuals and travel data, we predicted the timing of epidemics in each district of Pakistan during a large measles outbreak in 2012–2013 with over 30 000 reported cases. We combined these data with mobility data extracted from over 40 million mobile phone subscribers during the same time frame in the country to quantify the role of connectivity in the spread of measles. We investigate how different approaches could contribute to targeting vaccination efforts to reach districts before outbreaks started. While some prediction was possible, accuracy was low and we discuss key uncertainties linked to existing data streams that impede such inference and detail what data might be necessary to robustly infer timing of epidemics.

## Introduction

Despite considerable progress in reducing the global burden of measles infection over the last decades [1], measles remains a leading cause of childhood mortality in many low-income countries [2]. Measles control depends on the delivery of an effective and inexpensive vaccine that confers life-long protection. Challenges associated with vaccine delivery mean that spatial heterogeneity in coverage persists even in countries with otherwise effective vaccination programs allowing sporadic outbreaks to occur [3].

Characterising the features of control programs that allow measles outbreaks to occur is of clear programmatic importance. The underlying principles are well understood: the timing and magnitude of epidemics will be determined by seasonal fluctuations in transmission [4, 5] changing patterns of local susceptibility [6], extinction and the timing of introduction events that spark transmission chains [7]. Local susceptibility is defined by the history of vaccination coverage, exposure to natural infection and birth rates [6] and will determine whether measles goes locally extinct [6, 7]. Where measles is locally extinct, yet susceptibility is sufficiently high, generally as a result of the accumulation of unvaccinated births, outbreak risk depends on the probability of contact with an infected individual, shaped by patterns of spatial connectivity resulting from human travel [6].

In endemic pre-vaccination settings, regular, seasonal patterns of susceptible depletion and extinction and recolonisation occurred [6]. As countries introduced vaccination and eventually approached elimination, this regularity was lost and heterogeneities in susceptibility and movement were likely to play an increasingly important role in shaping dynamics [8]. Inference into the core drivers in these settings is more challenging: sparser incidence and greater immunity from vaccination amplify the role of stochasticity in dynamics. This occurs alongside increased uncertainty in the observation process since cases are rare. Theoretically, because both measles infection and vaccination are immunising, population susceptibility can be directly calculated by accounting for past births and subtracting natural infections and immunisation by vaccination. In reality, in most vaccinated population settings, uncertainty in historic case incidence [8], combined with spatially variable and often poorly documented vaccination coverage and doses [9] and complexities in disentangling vaccination from immunisation preclude this analysis. For example, many countries have inaccurate data on multiple doses, although this would help greatly illuminate the population at risk.

Settings with sufficiently detailed incidence data pre-vaccination allow estimates of another important variable for measles dynamics: the patterns of population connectivity underpinning re-introduction events [6]. Some populations experience disease-free periods because they are too small to sustain transmission chains in the face of stochastic extinction [10]. The duration of fade-outs (periods with zero detected cases) can be used to estimate the probability of measles being introduced to a community from another location. All else being equal, longer measles fade-outs are expected for less connected cities. Consequently, by accounting for seasonal fluctuations in measles transmission and susceptible accumulation and depletion [6], the length of disease-free periods can be used to estimate how connected each city is to the rest of the country [11]. Using this approach, it has been shown that for the UK, larger cities were more connected, i.e. experienced a higher probability of the arrival of an infected individual [11]. Similar approaches have been used to examine city connectivity in other pre-vaccination settings [12]. The need for accurately characterised patterns of susceptibility (challenging for reasons described above), in tandem with knowledge of the seasonal swing in the magnitude of transmission, complicate simultaneous estimation of these processes in vaccine-era settings.

Despite these challenges, understanding measles dynamics in the vaccine era is an increasingly important public health goal as progress is made towards elimination. While strengthening routine programs is broadly agreed to be of fundamental importance in moving forwards towards elimination [13], a related issue is identifying priority areas for further investment in control. If measles introduction risk can be adequately described, early warning systems could allow reactive vaccination programs to target districts or cities at highest risk of introduction when outbreaks occur. However, the degree to which health system data on vaccination coverage and incidence is of sufficient quality to allow the landscape of population susceptibility to be defined remains unclear [14]. Also unclear is the degree to which alternative sources of travel data (beyond directly using incidence data as described above) captures measles-relevant movement.

**Fig. 1.**
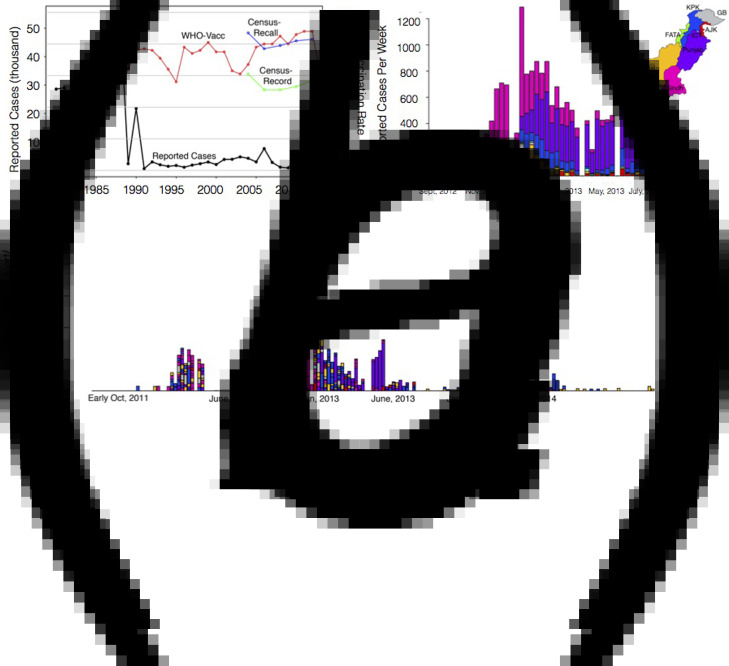
The country, province and district level measles data. (*a*) The country reported measles cases per year from 1980 to 2010 from the WHO (black line). Historically, the yearly number of cases has been steadily declining with <10 000 reported in recent years. In general, vaccination coverage has been increasing (red line), although the vaccination coverage estimate varies depending on the data source with census (blue, green lines) estimates often lower than WHO estimates (red). (*b*) In 2012–2013, a large measles outbreak was reported in Pakistan with over 30 000 suspected cases. The timing and province of these suspected cases are shown for the course of the epidemic. (*c*) Alert case data (see Materials and Methods) for each district coloured by the corresponding province from October 2011 to late November 2014 (province colours as in  1(b)).

To characterise the degree to which it was possible to anticipate measles spatial dynamics based on existing data-streams of vaccination coverage and mobility, we attempt to predict a large measles outbreak that took place in Pakistan in 2012–2013 and evaluate our prediction by comparing our estimated spatial and temporal outbreak risk to that observed via available incidence data from the outbreak. Pakistan is a country with a large (>185 million), mobile population with high birth rates (e.g. 31 births per 1000 population), as well as substantial spatial heterogeneity in measles vaccine coverage (see Supplementary Information). Prior to 2012, Pakistan had experienced a decade with very few large outbreaks of measles (Fig. 1a). In 2012–2013, however, the country experienced a large outbreak with more than 30 000 reported cases across the country (see Figs. 1b for outbreak cases for provinces, 1c for districts). While administrative measures of coverage – calculated as the number of doses divided by the target population size – are estimated to have increased from 60% to close to 90% since 2004 and the Pakistan Population and Health census estimates suggest that coverage has remained more or less stable at approximately 80% (by recall) or 60% (by vaccine record), implying that doses have been deployed, but may not be reaching their targets (see Materials and Methods).

Using an array of sources of measles data, vaccination coverage and travel, we estimated the key features defining spatial and temporal outbreak risk in Pakistan: population-level susceptibility and connectivity. We additionally compared multiple approaches to estimating human travel at the provincial and district spatial scale in Pakistan including an analysis of mobile phone data from approximately 40 million individual mobile subscribers over 7 months (1 June–31 December 2013), as well as a known human mobility model, i.e. the gravity model [15, 16]. We discuss what further evidence would be required to strengthen this inference and to move forward in validating the power of novel sources of travel data for early warning systems.

## Materials and methods

### Population and geographic data

Pakistan's large population is divided into eight provinces and areas; Balochistan, Punjab, Sindh, Khyber Pakhtunkhwa, Islamabad capital territory, Federally Administered Tribal Area (FATA), Gilgit-Baltistan and Azad Jammu & Kashmir. We obtained gridded population estimates and aggregated these data to the province and district levels (http://www.worldpop.org). The percentage of the population residing in urban and rural areas for each province and district were derived from Worldpop as well.

### Quantifying population-level susceptibility to measles

**Fig. 2.**
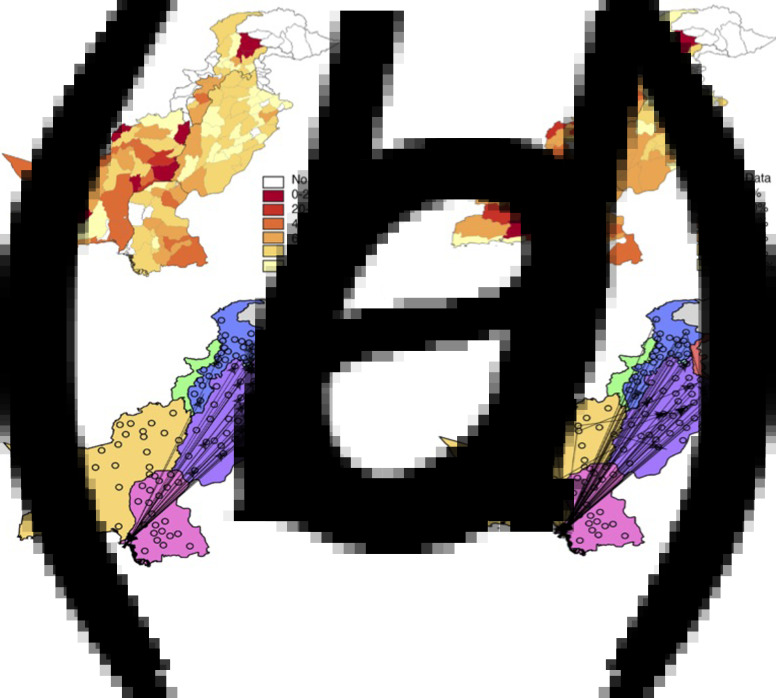
The vaccination and travel data for Pakistan. The reported vaccination coverage levels per district via the Pakistan Population and Health census (see Supplementary Information, Materials and Methods for details) in (*a*) 2004 and (*b*) 2012. Vaccination coverage is spatially heterogeneous with low coverage in Balochistan and KP provinces. Punjab has the highest coverage values, although even in this province many districts have coverage levels lower than 90%. In many locations, coverage has decreased from 2004 to 2012, most notably in Balochistan and the southern part of Punjab. (*c*) Using the mobile phone data or the (*d*) gravity model, travel between districts (black points) was quantified (see Materials and Methods). The top 0.5% of routes (based on the amount of travel) between districts is shown as an arrow. Travel estimated from the mobile phone data indicates large amounts of travel between Sindh province (pink) and Punjab (purple) with little travel to/from Balochistan (yellow).

National routine vaccination coverage from 1980 to 2010 was obtained from WHO and UNICEF estimates [3] (http://www.data.unicef.org/fckimages/uploads/immunization/pakistan.pdf). At a finer spatial scale, the Pakistan Population and Health census reported province and district level routine vaccination coverage values for urban and rural populations every 2 years (2004–2012) from household surveys (available in Supplementary Information, Fig. 1a, Table S2, Fig. 2a and b) across six of the provinces (Balochistan, FATA, Islamabad, KP, Punjab and Sindh, see Fig. S1). A child is considered vaccinated if they have a record of having received the measles-containing vaccine by 24 months of age.

In addition to the routine vaccination program, between 2007 and 2008, major supplementary immunisation activities (SIA) were conducted in the country with 66.5 million measles doses distributed, targeted to children ages 9 months–13 or 15 years [17] (http://www.who.int/immunization/monitoring_surveillance/data/en/). The ratio of doses deployed to the target population during these SIAs was >100%. However, a third of the cases reported in the 2012–2013 district level outbreak data were aged >5 years and thus would have had the opportunity to be immunised during the SIA (see Supplementary Fig. S5), suggesting less than complete coverage. We consequently assume coverage during SIAs to be 80% and verified that qualitative results were not sensitive to the exact value assumed (which may, in fact, be much lower, see e.g. [18]).

The data on routine and SIA coverage must then be combined with an estimate of the pattern of infection over age to estimate the fraction of the population that was immune at the time of the outbreak (2012). For each age, *a*, we first assumed that the proportion in that age cohort immunised by routine vaccination reflected the routine vaccination coverage reported for the year of their birth, using the finest spatial scale available in the relevant year (i.e. country, province, or district; for older individuals only country scale data was available). Next, for age cohorts that were within the target age range for SIAs, we increased this proportion to 80% if this value was larger than the reported value for routine immunisation, i.e. we assume complete overlap between vaccination campaigns and routine immunisation, as a conservative estimate that minimised our estimate of combined coverage. To account for the impacts of natural infection on susceptibility, we set the proportion of individuals protected by maternal immunity at age *a* to exp(−0.0375*a*) following [19]; and assumed that individuals acquired measles according to a constant hazard of infection of age. This was set such that in 1980, 90% of individuals were infected by age 20; to capture the fact that the hazard of infection will decline as vaccination coverage increases and case numbers fall, this rate was reduced according to the ratio of cases to have occurred over the period of low incidence between 1990 and 2012 (during the period of low incidence) relative to those in 1980 (as reported in [3]) yielding a *P*(infection by age *a*) = 1 − exp(−0.055 × *a*). This is clearly a simplification given the potential for erratic outbreaks, but as we were uncertain of the ability of the existing data to inform such spatial and temporal heterogeneity, we chose to use this relatively simple one-parameter approach. Modifying this rate does not alter the qualitative results of the analysis.

### Quantifying population travel

#### Mobile phone data

We analysed all voice-based, originated, call data records (CDRs) from 39 785 786 subscriber SIMs over a 7-month period, from 1 June till 31 December 2013 (see Fig. S8 for a geographic coverage map). These data are de-identified and are not considered human subjects data. These data were previously described elsewhere [16]. The tower level mobile phone data were aggregated to the corresponding district (admin level 2). On average, 15.2 million subscribers generated a record in the CDR per day. At the time of data acquisition, the mobile network operator was the second largest provider in Pakistan with approximately 25% of the market share. This source of information on connectivity is limited to national travel based on the operator data analysed and more detailed information about spatially variable subscribership was unavailable.

We focused on data originating from the six most populated provinces in Pakistan (Balochistan, Islamabad, FATA, KP, Punjab and Sindh) that also reported vaccination coverage estimates (Table S2, Fig. S1). Using previously developed methods, we estimated the daily location based on each subscriber's most frequently used mobile phone tower [16]. Every caller was assigned to their most frequently used base station/mobile phone tower on a given day. Using the inferred daily location of each caller based on the longitude and latitude of the most frequently used or most recently used routing tower, we measured daily travel between mobile phone towers relative to subscriber location on the previous day. Trips were aggregated to each district (Fig. 2c) based on the location of the origin and destination tower. The majority of travel connected Sindh and Punjab provinces (see Fig. 2c) along the major national highway (see Fig. S4 for a major roadmap). For further analyses designed to explore the degree to which this data capture measles relevant introduction pressures, we also aggregated all incoming trips to create a single measure of incoming travel.

#### Gravity model

Although we were able to directly measure mobility patterns using mobile phone data, in many instances these data may not be available, particularly during an outbreak. In lieu of these data, traditional sources of travel data, such as a national census, may be used. However, in Pakistan, these data are unavailable, leading us to use a baseline spatial interaction model as a basis of comparison for mobile phone travel, i.e. the gravity model. This type of model using the below framework is possible to quantify in lieu of these other data sources. Following a naïve gravity model specification, the amount of travel between districts *i* and *j* of population size *N*_*i*_ and *N*_*j*_ (with populations obtained from Worldpop) is equal to *N*_*i*_*N_j_* /distance(*i,j*), [20] and distance reflects Euclidean distance between district centroids (see Fig. 2d). Previous analyses have found that the effect of both source and destination population sizes scales with an exponent slightly larger than unity [21]; and that there was either no effect of distance [21]; or linear scaling as in the relationship above [22]. Since it was not clear exactly what scaling might be appropriate in this setting and since qualitative patterns were invariant for small changes in exponents on the components of the numerator and it seemed reasonable to assume some effect of distance, we retained this simple framing.

### Available measles data

We used three sources of measles data: national reported cases, province outbreak cases and district alert data.

#### National level historical data

Yearly countrywide measles cases have been reported to the WHO since 1980 (Fig. 1a); and are available in the Supplementary Information (Fig. 1a).

#### Province level case data

During the 2012–2013 measles outbreak, the number of cases per province were reported in the Weekly Epidemiological Reports published by the National Institute of Health, Islamabad and the World Health Organisation, as part of a weekly disease system that provides highlights of their early warning disease system and responses to these warnings across the country. These data were digitised (mid-September 2012– early September 2013) (Fig. 1b, Supplementary Information for data) and geolocated to the six most populated provinces (Balochistan, FATA, Islamabad, Khyber Pakhtunkhwa (KP), Punjab and Sindh).

#### Alert district level data

The Weekly Epidemiological Reports also publishes measles-related ‘outbreaks’ and ‘alerts’. Alerts are any report of a suspected case of measles in any reporting health facility in the week. ‘Outbreaks’ are defined as five or more related cases from a single alert. We analysed the data of reports that met the outbreak classification but refer to these cases as alert cases to avoid confusion with the 2012–2013 outbreak-related case data. These reports were digitised from week 40, 2011 to week 48, 2014 (see Fig. 1c, Supplementary Information). The location (up to the reported district), date, the age of the cases (<5 or 5+), gender and a number of measles cases associated with each alert (five or more associated cases) were analysed. Importantly, surveillance system capacity is likely to vary across the country and this will result in a distorted view of the pattern of outbreaks. Our focus here was on evaluating the potential for using existing data-streams to project future outcomes, so we did not investigate this further, but an interesting direction for future research might be to evaluate correlation between vaccination coverage, case reporting and other economic or development indices across districts to develop an index for surveillance capacity across the country.

### Combining the landscape of susceptibility with mobility to define outbreak risk

We predicted the timing of measles outbreaks from the available data on susceptibility and mobility and evaluated this prediction using the available incidence data. In a district that currently has no measles cases, a new outbreak will spark if there is contact with an infected individual from elsewhere and if both the size of the susceptible pool and the magnitude of transmission are sufficient to allow the epidemic to take off [6]. The discrete time hazard of the outbreak, *h(t,j),* for time *t* and location *j,* can be estimated as:

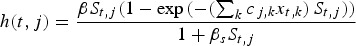

where *β* is a transmission coefficient; *S*_*t*,*j*_ is the proportion of the population that is susceptible in location *j* calculated as described above; *c*_*j*,*k*_ reflects mobility from location *k* to location *j*; and *x*_*t*,*k*_ is the fraction of the population that is infected in location *k*. We can compare outcomes for connectivity matrices *c*_*j*,*k*_ that (i) assume equal connectivity in all locations, (ii) follow the gravity model and (iii) follow the mobile phone data.

Estimating the magnitude of transmission *β* is intractable given the measles incidence data available for Pakistan and we thus assume a value reflecting *R*_0_ = 15 as observed for measles in many settings [6]; changing this does not change the qualitative results of our analysis. We assumed that transmission did not vary spatially, or seasonally, despite the fact that seasonal fluctuations in transmission linked to seasonal changes in population aggregation are a general feature of measles [11, 23]. However, if the seasonal variation is similar across the country, as might be the case if school terms are the key driver [11], this will mitigate the impact on our inference.

We initiated our analysis assuming that a case was present in each of the districts where alert data reported (starting the analysis during the week of 22 September 2013) and then projected measles incidence by simulating a stochastic process of introduction into districts where measles was currently extinct according to the hazard defined above:

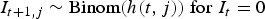


In districts where measles had emerged, we simulated deterministic dynamics, including both transmission and susceptible depletion, according to:

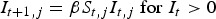


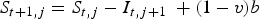

where *β* = 15 as above, *v* is the vaccination coverage in each district, *b* is the approximate birth rate ( ~28 per 1000 per year) and we use a time step of 2-weeks, the generation time of measles.

As neither the gravity model nor the mobile phone data provide absolute measures of individuals moving (e.g. for the mobile phone data, we are constrained to subscribers), to create predictions that resemble the observed patterns of the timing of measles outbreaks across districts first requires rescaling the mobility matrices. Accordingly, we first simulated time-series of measles for the metapopulation of districts in Pakistan, across a gradient of scalars multiplying each of the three connectivity matrices and introducing the resulting *c*_*j*,*k*_ into the equation for *h*(*t*,*j*) defined above. We repeated the simulation 100 times for each value of the scalar. We then selected the scalar that resulted in the minimum sum of squares difference between the observed and predicted timing or biweek in which measles was first seen, in each district across the 100 simulations for each scalar (see Fig. S6).

**Fig. 3.**
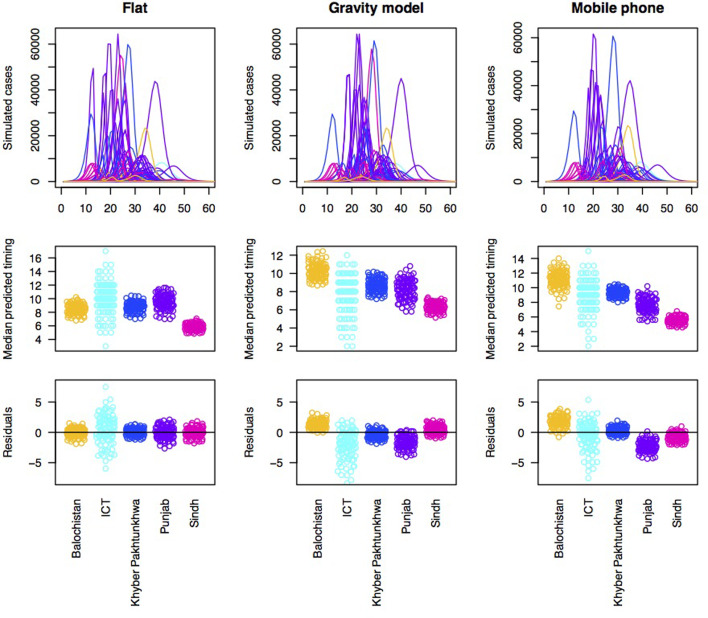
Simulations of the timing of outbreaks for three connectivity models. Using a flat connectivity, gravity model and mobile phone data we simulated the time series of incidence for 60 biweeks for each district. One example simulation of time series of incidence for each model (top row), the median predicted the timing for each district across provinces for 100 simulations (middle row) and residuals for each district around a regression linking the median of 100 simulations to the observed timing organised by province (bottom row). This latter is evaluated to explore the degree to which the evidence suggests that susceptibility and/or connectivity might be over or under-estimated in particular provinces. For both the gravity model and the mobile phone connectivity-based model, there is a suggestion that Balochistan is more delayed in the simulations as expected; and Punjab is earlier than expected.

With each type of connectivity matrix scaled to most closely reflect the observed pattern of outbreaks, we subsequently simulated measles dynamics across the metapopulation of Pakistan and compared the timing of measles outbreaks in each district to the timing of measles outbreaks observed in the data (Fig. 3). In order to better understand how and where prediction failed, we plotted the pattern of residuals across provinces to see if districts from particular provinces tended to be delayed or earlier in the simulation relative to the observed. Delays could be attributed to under-estimation of susceptibility or under-estimation of connectivity to districts within this province; and vice versa.

## Results

Vaccination coverage varied by province and between rural and urban areas. For example, Islamabad, the capital province, consistently reported the highest routine vaccination values for both rural (average over the time frame: 92.2%) and urban (average over the time frame 89.8%) populations. By contrast, Balochistan, a primarily rural province (10% of the population lives in urban areas) in the western part of the country, had the lowest vaccination values for both urban (average over the time frame: 77.4%) and rural areas (average over the time frame: 47.8%) (Fig. S1). Nationally, in the years following 2005, vaccination coverage values were all below the 90% theoretical threshold for measles elimination required even for a relatively low estimate of *R*_0_ = 10 (see Fig. 1a). This translates into a considerable risk of outbreaks throughout this between 2005 and 2012, particularly given large birth cohorts. The SIA in 2007–2008 may have been partly responsible for the fact that outbreaks were not observed. However, evidence for lower coverage in this campaign [24] suggests a role for other aspects. Candidates include heterogeneity in vaccination coverage, coupled with incomplete mixing across the country, which could interrupt disease circulation and delay the outbreak, even though herd immunity had not been established.

At smaller spatial scales (Fig. 2a and b, Supplementary Information for data), vaccination coverage ranged from an average of 0–99% (mean: 79%, standard deviation: 19%) in urban areas, 3–99% (mean: 72%, standard deviation: 20%) in rural areas. By 2010, only 4% of the districts had >90% vaccination coverage [16] (mean vaccination coverage for districts: 73% in 2010, 2012, see Supplementary Information for data, Fig. S1, Fig. 2a and b) and in many parts of the country, vaccination coverage values have been decreasing [24] (Fig. 2a and b, Supplementary Information for data) with possible explanations including failures in the supply chain, difficulty reaching nomadic populations and an increased reliance on non-routine vaccination campaigns [24]. Even in areas with some of the highest coverage levels (e.g. Punjab) vaccination coverage levels alone suggest the potential for outbreaks (Fig. S1). Districts in Balochistan and Sindh, in particular, experienced strikingly high risk (vaccination coverage of only 58% and 70%, respectively in 2010). Again, vaccination heterogeneity and contact heterogeneity are likely to have played a role in delaying contacts between infected and susceptible individuals, thus delaying measles spread and resulting in the large observed outbreak.

Using these data, we estimated population-level susceptibility combining vaccination and natural immunity (Supplementary Information). Overall, province level susceptibility varied between 9% (Islamabad) and 14% (Balochistan) (see Fig. S2). District level susceptibility had a wide range of values 7% – 20% (mean 11%) (see Fig. S3).

To estimate potential routes of measles introduction events, we first analysed mobile phone data from mobile phone subscribers between 1 June and 31 December 2013, using methods previously described (see Materials and Methods, general travel results found in [16]). The sampled population took an estimated 53 000 average number of trips between provinces (300 average number of trips between districts) per day. The majority of travel followed a southwest – northeast corridor along the major highway to/from Punjab and Sindh provinces (see Fig. 2c, Fig. S4 for a major roadmap). Across Pakistan, we also identified a large amount of travel between districts, occurring predominantly within provinces – but there is also considerable travel between districts in the Punjab and Sindh provinces and districts in other provinces (see Fig. 2c). In comparison, the amount of travel estimated from the gravity model is primarily trips to Punjab province (see Fig. 2d). For districts, the gravity model estimates a more diffuse pattern of travel with less within-province travel than the mobile phone data suggests.

We evaluated the degree to which population-level susceptibility and three estimates of connectivity (mobile-phone derived, gravity model derived and uniform, i.e. assuming equal connectivity between all districts of Pakistan) could describe the spatial and temporal patterns of 2012–2013 measles outbreak (see Fig. 1b, Fig. S5 and Supplementary Information for outbreak data). Cases were reported in all provinces, with the majority in Punjab (8529 cases) and Sindh (6684 cases), where 75% of the population resides (see Fig. 1b). The earliest reported cases were in Sindh, Azad Kashmir (AJK) and Balochistan provinces (see Fig. 1b). Sindh is the second most populous province whereas AJK and Balochistan are predominantly rural (Fig. 1b). The outbreak peaked latest in Punjab province.

We would expect early outbreaks in locations with a large amount of incoming travel and high susceptibility since these locations are the most at risk of an introduction event (incoming travel) and subsequent outbreak (susceptibility). This would suggest that Punjab, Sindh and KP should have the earliest outbreaks with Islamabad unlikely to have an early outbreak (see Fig. 1b, Fig. S2, Supplementary Information). Although Sindh and KP do have cases early on (see Fig. 1b), surprisingly the outbreak does not reach Punjab until much later. When we consider the district level alert data (if a clinic reported at least five suspected linked cases of measles based on the clinical presentation) from 2011 to 2014 (Fig. 1c), that includes the large 2012–2013 outbreak, the relationship between the timing of reported cases, district level susceptibility and incoming travel is also at odds with expectations. Although the earliest district alerts occurred in Sindh, followed later by Punjab, as broadly expected based on vaccination coverage and connectivity (Fig. 1c, Fig. S2 and S3, Supplementary Information), a regression with magnitude of connectivity and coverage fitted as covariates explains very little of the variation in timing of outbreaks (adjusted *R*^2^ = 0.188, see Table S1). Beyond potential biases in estimates of the timing of outbreaks resulting from variability in health facility reporting, this mismatch may be because these analyses do not take into the underlying transmission dynamics.

Accordingly, to formally evaluate the effect of susceptibility and connectivity on the distribution of delays until case occurrence in the alert data, we simulated the pattern of outbreaks under an array of connectivity patterns (Fig. 3, top row). All models broadly predict that outbreaks should start in Sindh, followed by Punjab, followed by KPK or ICT; Balochistan is generally anticipated to come last (Fig. 3, 2nd row). Yet, the province data indicate much earlier outbreaks in Balochistan provinces (Fig. 3, 3rd row), with Punjab coming last, indicative of a mismatch across the different data sources, even when dynamical interactions are accounted for.

**Fig. 4.**
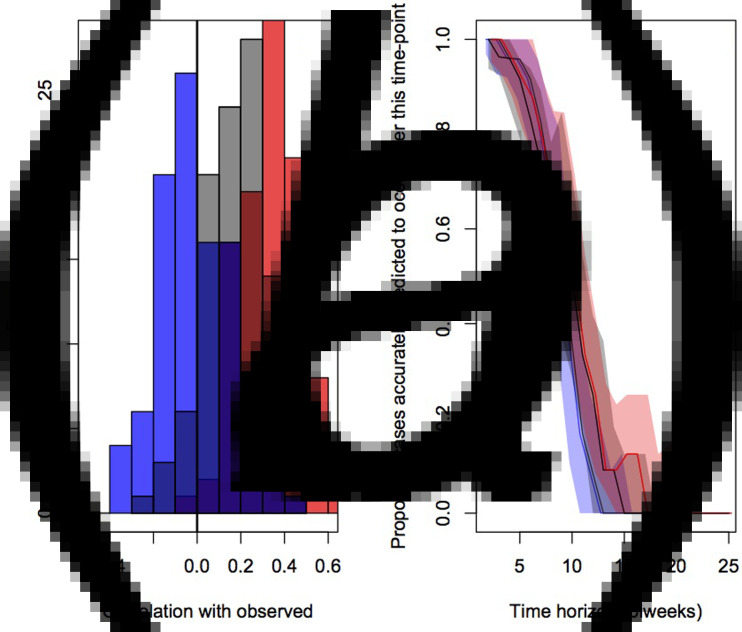
Comparison of prediction of outbreak order for three connectivity models (flat in blue, gravity models in grey and model phone data in red) showing (*a*) the distributions of correlations between the observed and expected; and (*b*) proportion of districts accurately predicted to occur in the future for varying delays (colours as in previous), relevant for planning interventions.

To evaluate the accuracy and bias of projections of the timing of the different connectivity matrices, we compared the correlation between the predicted timing of outbreaks in districts and the observed timing across 100 simulations (Fig. 4a). This indicated the lowest correlation on average for the assumption of equal connectivity between all districts (median correlation of −0.01, quartiles of −0.09 and 0.09), intermediate correlation for the gravity model districts (median correlation of 0.12, quartiles of 0.02 and 0.25) and highest correlation for the mobile phone data (median correlation of 0.35, quartiles of 0.21 and 0.45).

The public health application of the analyses described here will be in shaping the strategic deployment of vaccination campaigns in order to mitigate outbreaks by arriving early. We therefore also evaluated the fraction of cases appropriately predicted to occur after a chosen time-horizon for each of the three connectivity matrices (Fig. 4b). Early on, all three do equally well and have good concordance with the data (expected since most locations have yet to experience an outbreak); after approximately the 6th month, the mobile phone data provides the best projection. It is important to note, however, that even for this ‘best’ model, accuracy is still low.

## Discussion

Measles control efforts are strengthening globally and more and more countries are approaching elimination [8]. Given persisting inadequacies in vaccination programs, the trajectory towards elimination may result in a period of low incidence that allows accumulation of susceptible individuals [25], as not everyone is vaccinated, but the risk of infection of unvaccinated individuals is reduced, as the infection is rare, so that these unvaccinated individuals may age without acquiring infection until re-introduction of the pathogen starts an outbreak. Large, late age outbreaks following such a ‘honey-moon’ period have been observed in many parts of the world (e.g. Burkina Faso 2009 [26], Cameroon 2008–2009 [27], Zambia 2010–2011 [28], Malawi 2010 [29], France 2008–2011 [30]), often demonstrating detectable spatial spread [29]. Preparing for a disease outbreak by either planning reactive vaccination or increasing vaccination coverage preemptively at the local scale is a key way to reduce mortality and morbidity in such measles honeymoon outbreak settings [29]. Early interventions will have the greatest impact on measles morbidity and mortality [31] and substantial numbers of cases may be averted even if the outbreak has already started [32]. It can take several months to organise a vaccination campaign (around 2 months, for example, during the 2010 outbreak in Malawi [29]) and any sources of information that could allow for earlier warning and targeting of investment in campaigns would be a powerful improvement.

The potentially considerable public health benefits of anticipating measles outbreaks [32] lead us to explore our ability to predict the timing of outbreaks based on available epidemiological data, combined with estimates of human mobility. Given the data available, our framework could predict more than half the districts where outbreaks would start after ~4 months in the future; and, assuming mobile phone derived connectivity, could predict up to around 20% of outbreaks that would start after ~6 months in the future (Fig. 4b). While this provides an interesting starting point of potential relevance for vaccination efforts, accuracy is still low and the range of predictions is wide (Fig. 4b). Further, although ordering of district outbreaks itself is likely to be of value for prioritisation of control efforts in resource-poor settings and this characteristic will be tractable with only information on susceptibility and connectivity, identifying the magnitude of timing (e.g. time in months till the outbreak starts) will require more information. Here, we leveraged simulations based on the known trajectory of outbreaks to predict the timing (Fig. S6) and this data will not be available in settings where vaccine targeting must be deployed.

Interpreting our results is complicated by the fact that all three sources of information that we build upon each are subject to potential biases, which could have contributed to a mismatch between our simulations and the observed patterns. These include misspecification of patterns (i) of incidence; (ii) of susceptibility; and (iii) of connectivity.

First, measles case data are only available at broad spatial resolution (i.e. the province level), which could obscure the order of emergence. While ‘alert data’ are available at finer spatial scales (the district level), its potentially erratic nature might obscure the nuance of the order of emergence. The ideal source of data for this analysis would be measles case data at the district level over multiple years, allowing inference into patterns of connectivity from the measles data itself (i.e. as in [22, 33]). Despite these issues, it remains possible that the ordering of outbreaks indicated by the case and alert data is accurate: in particular, the mismatch identified suggests that outbreaks in Sindh and Balochistan precede outbreaks in Punjab, despite the fact that Punjab, a resource-rich province, is likely to have a well-performing surveillance system and therefore, if anything, one which should be reporting outbreaks earlier if there were cases. This suggests that the quality of the measles data alone may not be the main issue.

Second, predicting the timing of outbreaks requires unbiased estimates of susceptibility that will be based on fine-scale estimates of vaccination coverage [9]. Generally, spatial fluctuations in reporting are a major challenge to obtaining this information and cross-validation between different data sources (for example, evaluating whether estimates of vaccination coverage, incidence, the age of infection follow expectations across locations) will be important to strengthen our knowledge of variability in patterns of measles vaccination coverage. Here, we benefited from an extensive household survey based data-source (rather than the usually available administrative data). However, these data necessarily reflect a subsample of the population and are only available in a subset of years, so uncertainties are likely to persist. Additionally, vaccination alone will not be the only source of immunity. Despite the strikingly large caseload studied here, suggestive of a post-honeymoon outbreak [25, 34], various other threads of evidence indicated that solid herd immunity had not been established, thus pointing to a role for persistent erratic local chains of transmission in shaping spatial patterns of susceptibility, as well as emphasising a more important immediate investment case in bolstering immunisation programs. Although we can only address this question at a relatively broad scale (districts) and cannot address the fact that altering any driver (susceptibility, connectivity, etc.) will modify the whole pattern of timing given the tightly interdependent nature of the components, our investigation suggests that, if the connectivity matrices are accurate, districts in Balochistan might be over-estimating vaccination coverage. Indeed, increasing susceptibility in this province within our simulation framework moves the timing of outbreaks in Balochistan to closer to the observed timing (Fig. S7).

Finally, understanding the timing of measles outbreaks requires being able to capture connectivity of human populations. Although technically, the spatial scale of mobile phone data or the gravity model could be very fine, we were constrained by the scale of the incidence data to focusing at the district level and this might have obscured the patterns we were trying to titrate. Alternatively, it might be the case that neither measure appropriately reflects mobility of key individuals for measles introductions. Gravity-like models are likely to yield an oversimplified description of human movement [15], but mobile phone data may also be biased by ownership and call frequency, particularly given that mobile phone owners are more likely to be adults and in Pakistan, children are the highest burden of measles (although analyses in Kenya suggest that children are unlikely to travel without an adult [35]). In addition, the mobile phone data only covered 7 months of the year possibly missing seasonal differences in travel. A longer time series of mobility data could help address these concerns. Additionally, ever increasing mobile phone ownership could further strengthen the precision of this data-stream in calibrating patterns of human mobility.

Additionally, neither model of connectivity deployed tackled the issue of international travel patterns, despite the possibility of introductions from outside of Pakistan being an important consideration, particularly from neighbouring Afghanistan where vaccination coverage is low (e.g. vaccination coverage was ~60% in 2013), measles cases are regularly reported and which represents the largest proportion of the migrant population in Pakistan (57%) [36]. Data on cross-border importation is difficult to obtain, although the politically closed situation in Pakistan makes importations from China, India and Iran unlikely since the majority of its borders are highly restricted. The impact of international travel will vary by connectivity and current measles epidemiology in neighboring countries.

To conclude, our analysis suggests that it is possible to anticipate the ordering of outbreaks across districts following a honeymoon period if data on susceptibility and connectivity is adequate. Furthermore, given a priori expectations for likely uncertainties in the data available (e.g. in susceptibility, but also reporting of incidence and connectivity), our model framework could be used to systematically explore their role in modulating timing and the mismatch between simulated and observed dynamics (as in Fig. S2 to explore the role of susceptibility in Balochistan). Such analyses, combined with analyses across further settings, especially settings where case data is routinely reported in a spatially resolved and timely fashion could delineate the limits of predictability within this framework and thus its potential to guide vaccination targeting within the current landscape of measles elimination efforts.

## Supplementary material

For supplementary material accompanying this paper visit http://doi.org/10.1017/S0950268818001449. click here to view supplementary material
