# Supplementary material for: Measles outbreak risk in Pakistan: exploring the potential of combining vaccination coverage and incidence data with novel data-streams to strengthen control
Source: Epidemiol Infect. 2018 Jun 4;146(12):1575–83. doi: 10.1017/S0950268818001449 (PMC6090714; doi:10.1017/S0950268818001449)
Supplement: Supplementary file 1 [file S0950268818001449sup001.docx]

**Measles outbreak risk in Pakistan: exploring the potential of combining health system data with novel data-streams to strengthen control**

Amy Wesolowski^1^, Amy Winter^2^, Andrew J. Tatem^3,4,5^, Taimur Qureshi^6^, Kenth Engø-Monsen^6^, Caroline O. Buckee ^7,8^, Derek A.T. Cummings^9, 10^, C. Jessica E. Metcalf^2,11^

^1^ Department of Epidemiology, Johns Hopkins Bloomberg School of Public Health, Baltimore, USA.

^2^ Department of Ecology and Evolutionary Biology, Princeton University, Princeton, USA.

^3^ Department of Geography and Environment, University of Southampton, Southampton, UK.

^4^ Fogarty International Center, National Institutes of Health, Bethesda, USA.

^5^ Flowminder Foundation, Stockholm, Sweden.

^6^ Telenor Research, Oslo, Norway.

^7^ Department of Epidemiology, Harvard T.H. Chan School of Public Health, Boston, USA.

^8^ Center for Communicable Disease Dynamics, Harvard T.H. Chan School of Public Health, Boston, USA.

^9^ Department of Biology, University of Florida, Gainesville, USA.

^10^ Emerging Pathogens Institute, University of Florida, Gainesville, USA.

^11^ Woodrow Wilson School, Princeton University, Princeton, USA.

correspondence: awesolowski@jhu.edu

**Supplementary Information**

**Data supplement**

Below we outline the data provided as part of a data supplement. For all files, missing values are left blank.

DistrictProvVaccCov.csv

The vaccination coverage estimates from the Pakistan Population and Health Census for each reporting district. Vaccination coverage values are broken down by Urban, Rural and All. Recorded (Measles-Record-YEAR) and recall (Measles-YEAR), when reported are available.

Reconstruct_District_PropSuscep.csv

The reconstructed (see Materials and Methods) proportion of the population susceptible and the percentage of the population considered urban per district are shown.

Reconstruct_Prov_PropSuscep.csv

The reconstructed (see Materials and Methods) proportion of the population susceptible, the percentage of the population considered urban, and population per province are shown.

District_WeeklyMeaslesOutbreak_FullData.csv

The weekly number of measles alerts per week from Week 1, 2012 till Week 48, 2015 for all reporting districts.

Prov_WeeklyMeasles_FullData.csv

The number of alerts, outbreaks, cases, and deaths reported per province per week from Week 20, 2012 till Week 34, 2013.

*Pakistan Geography*

Pakistan is located in South Asia and borders India, Afghanistan, Iran, and China. It is divided into eight provinces; that are further subdivided into 128 (plus 7 tribal agencies) districts (equivalent to admin unit 2 from 2008, see Figure 1B). Punjab (54% of the population) is the most populated province along the eastern border with India and includes many cities such as Lahore, Rawalpindi, and Faisalabad. Sindh (22%) is the second most populous province and includes the largest city, Karachi, along the southern coast.

*Measles Data in Pakistan*

We used three sources of measles data: national reported cases, province outbreak cases, and district alert data.

*National level historic data*: Yearly countrywide measles cases have been reported to the WHO since 1980; and are available in the Supplementary Information (Figure 1A). Totals range from ~600 cases reported in 1991 to ~55,500 cases reported in 1988 (average from 1980 – 2010: 11,800 cases). More recently (from 2000 to 2010), the number of reported cases is fewer than 10,000 (mean number of cases reported from 2000 to 2010: 3,500 cases).

*Province level case data*: During the 2012-2013 measles outbreak, the number of cases per province were reported in the Weekly Epidemiological Reports published by the National Institute of Health, Islamabad and the World Health Organization, as part of a weekly disease system that provides highlights of their early warning disease system and responses to these warnings across the country. These data were digitized (mid September, 2012- early September, 2013) (see Figure 1B, Supplementary Information for data). In total, 20,674 cases were geo-located to one of eight provinces (provinces: Azad Jammu & Kashmir (AJK), Balochistan, FATA, Gilgit-Baltistan (GB), Islamabad, Khyber Pakhtunkhwa (KP), Punjab, and Sindh). In order to relate these data to the vaccination data, we focused on the six most populated provinces of the country: Balochistan, FATA, Islamabad, KP, Punjab, and Sindh.

*Alert district level data*: The Weekly Epidemiological Reports also publishes measles related ‘outbreaks’ and ‘alerts’. Alerts are any report of a suspected case of measles in any reporting health facility in the week. ‘Outbreaks’ are defined as five or more related cases from a single alert. We analyzed the data of reports that met the outbreak classification, but refer to these cases as alert cases to avoid confusion with the 2012-2013 outbreak related case data. These reports were digitized from week 40, 2011- week 48, 2014 (see Figure 1C, Supplementary Information). The location (up to the reported district), date, age of the cases (<5 or 5+), gender, and number of measles cases associated with each alert (5 or more associated cases) were analyzed. In general, between 85-90 districts (out of 128 total districts) from across Pakistan report weekly. It should be noted that although this is the most finally spatially resolved data available for understanding measles dynamics in Pakistan, these alert cases represent a subset of the reported case data and are likely spatially biased based on the location of reporting clinics. In total, we digitized and analyzed ~7,000 alert related cases.

**Quantifying population level susceptibility to measles**

In addition to the routine vaccination program, between 2007-2008, major supplementary immunization activities (SIA) were conducted in the country with 66.5M measles doses distributed, targeted to children ages 9M-13Y or 15Y [17] (<http://www.who.int/immunization/monitoring_surveillance/data/en/>). The ratio of doses deployed to the target population during these SIAs was greater than 100%. However, a third of the cases reported in the 2012-2013 district level outbreak data were aged greater than 5 years, and thus would have had the opportunity to be immunized during the SIA (see Supplementary Figure S5), suggesting less than complete coverage. We consequently assume coverage during SIAs to be 80%, and verified that qualitative results were not sensitive to the exact value assumed (which may in fact be much lower, see e.g., [18]).

The data on routine and SIA coverage must then be combined with an estimate of the pattern of infection over age to estimate the fraction of the population that was immune at the time of the outbreak (2012). For each age, *a*, we first assumed that the proportion in that age cohort immunized by routine vaccination reflected the routine vaccination coverage reported for the year of their birth, using the finest spatial scale available in the relevant year (i.e., country, province, or district; for older individuals only country scale data was available). Next, for age cohorts that were within the target age range for SIAs, we increased this proportion to 80% if this value was larger than the reported value for routine immunization, i.e., we assume complete overlap between vaccination campaigns and routine immunization, as a conservative estimate that minimized our estimate of combined coverage. To account for the impacts of natural infection on susceptibility, we set the proportion of individuals protected by maternal immunity at age *a* to exp(-0.0375*a*) following [19]; and assumed that individuals acquired measles according to a constant hazard of infection of age. This was set such that in 1980, 90% of individuals were infected by age 20; to capture the fact that the hazard of infection will decline as vaccination coverage increases and case numbers fall, this rate was reduced according the ratio of cases to have occurred over the period of low incidence between 1990 and 2012 (during the period of low incidence) relative to those in 1980 (as reported in [3]) yielding a 𝑃(infection by age 𝑎) = 1 − 𝑒𝑥𝑝(−0.055 × 𝑎). This is clearly a simplification given the potential for erratic outbreaks, but as we were uncertain of the ability of the existing data to inform such spatial and temporal heterogeneity, we chose to use this relatively simple one-parameter approach. Modifying this rate does not alter the qualitative results of the analysis.

**Quantifying population travel**

*Mobile phone data:* We analyzed all voice-based, originated, call data records (CDRs) from 39,785,786 subscriber SIMs over a seven-month period, from June 1 till December 31, 2013 (see Figure S8 for a geographic coverage map). These data are deidentified and are not considered human subjects data. These data were previously described elsewhere [16]. The tower level mobile phone data were aggregated to the corresponding district (admin level 2). On average, 15.2 million subscribers generated a record in the CDR per day. At the time of data acquisition, the mobile network operator was the second largest provider in Pakistan with approximately 25% of the market share. This source of information on connectivity is limited to national travel based on the operator data analyzed.

We focused on data originating from the six most populated provinces in Pakistan (Balochistan, Islamabad, FATA, KP, Punjab, and Sindh) that also reported vaccination coverage estimates (Table S2, Figure S1). Using previously developed methods, we estimated the daily location based on each subscriber’s most frequently used mobile phone tower [16]. Every caller was assigned to their most frequently used base station/mobile phone tower on a given day. Using the inferred daily location of each caller based on the longitude and latitude of the most frequently used or most recently used routing tower, we measured daily travel between mobile phone towers relative to subscriber location on the previous day. Trips were aggregated to each district (Figure 2C) based on the location of the origin and destination tower. The majority of travel connected Sindh and Punjab provinces (see Figure 2C) along the major national highway (see Figure S4 for a major road map). For further analyses designed to explore the degree to which this data captures measles relevant introduction pressures, we also aggregated all incoming trips to create a single measure of incoming travel.

*Gravity model:* Although we were able to directly measure mobility patterns using mobile phone data, in many instances these data may not be available, particularly during an outbreak. In lieu of these data, traditional sources of travel data, such as a national census, may be used. However, in Pakistan, these data are unavailable, leading us to use a baseline spatial interaction model as a basis of comparison for mobile phone travel, i.e., the gravity model. Following a naïve gravity model specification, the amount of travel between districts *i* and *j* of population size *N_i_* and *N_j_* is equal to *N_i_N_j_* /distance(i,j), [20] and distance reflects Euclidean distance between district centroids (see Figure 2D). Previous analyses have found that the effect of both source and destination population sizes scales with an exponent slightly larger than unity [21]; and that there was either no effect of distance [21]; or linear scaling as in the relationship above [22]. Since it was not clear exactly what scaling might be appropriate in this setting, and since qualitative patterns were invariant for small changes in exponents on the components of the numerator, and it seemed reasonable to assume some effect of distance. We retained this simple framing.

**Combining the landscape of susceptibility with mobility to define outbreak risk**

We predicted the timing of measles outbreaks from the available data on susceptibility and mobility and evaluated this prediction using the available incidence data. In a district that currently has no measles cases, a new outbreak will spark if there is contact with an infected individual from elsewhere, and if both the size of the susceptible pool and the magnitude of transmission are sufficient to allow the epidemic to take off [6]. The discrete time hazard of outbreak, *h(t,j),* for time *t* and location *j,* can be estimated as:

*h*(*t*,*j*) = [*βS_t_*_,_*_j_* (1-exp(-(Σ_k_*c_j_*_,_*_k_ x_t_*_,_*_k_*)*S_t_*_,_*_j_*))]/[1+*β_s_ S_t_*_,_*_j_*]

where *β* is a transmission coefficient; *S_t_*_,_*_j_* is the proportion of the population that is susceptible in location *j* calculated as described above; *c_j_*_,_*_k_* reflects mobility from location *k* to location *j*; and *x_t_*_,_*_k_* is the fraction of the population that is infected in location *k*. We can compare outcomes for connectivity matrices *c_j_*_,_*_k_* that i) assume equal connectivity in all locations, ii) follow the gravity model, and iii) follow the mobile phone data.

Estimating the magnitude of transmission *β* is intractable given the measles incidence data available for Pakistan, and we thus assume a value reflecting R_0_=15 as observed for measles in many settings [6]; changing this does not change the qualitative results of our analysis. We assumed that transmission did not vary spatially, or seasonally, despite the fact that seasonal fluctuations in transmission linked to seasonal changes in population aggregation are a general feature of measles [12, 23]. However, if seasonal variation is similar across the country, as might be the case if school terms are the key driver [12], this will mitigate the impact on our inference.

We initiated our analysis assuming that a case was present in each of the districts where alert data reported (starting the analysis during the week of 22 Sept, 2013), and then projected measles incidence by simulating a stochastic process of introduction into districts where measles was currently extinct according to the hazard defined above:

*I_t+1_*_,_*_j_* ~ Binom(*h*(*t*,*j*)) for *I_t_* = 0

In districts where measles had emerged, we simulated deterministic dynamics, including both transmission and susceptible depletion, according to:

*I_t+1_*_,_*_j_* = *β S_t_*_,_*_j_* *I_t_*_,_*_j_* for *I_t_* > 0

*S_t+1_*_,_*_j_* = *S_t_*_,_*_j_* - *I_t_*_,_*_j+1_* + (1-*v*)*b*

where *β* =15 as above, *v* is the vaccination coverage in each district, *b* is the approximate birth rate (~ 28 per 1000 per year) and we use a time step of two-weeks, the generation time of measles.

As neither the gravity model nor the mobile phone data provide absolute measures of individuals moving (e.g., for the mobile phone data, we are constrained to subscribers), to create predictions that resemble the observed patterns of timing of measles outbreaks across districts first requires rescaling the mobility matrices. Accordingly, we first simulated time-series of measles for the metapopulation of districts in Pakistan, across a gradient of scalars multiplying each of the three connectivity matrices, and introducing the resulting *c_j_*_,_*_k_* into the equation for *h*(*t*,*j*) defined above. We repeated the simulation 100 times for each value of the scalar. We then selected the scalar that resulted in the minimum sum of squares difference between the observed and predicted timing, or biweek in which measles was first seen, in each district across the 100 simulations for each scalar (see Figure S6).

With each type of connectivity matrix scaled to most closely reflect the observed pattern of outbreaks, we subsequently simulated measles dynamics across the metapopulation of Pakistan, and compared the timing of measles outbreaks in each district to the timing of measles outbreaks observed in the data (Figure 3). In order to better understand how and where prediction failed, we plotted the pattern of residuals across provinces to see if districts from particular provinces tended to be delayed or earlier in the simulation relative to the observed. Delays could be attributed to under-estimation of susceptibility or under-estimation of connectivity to districts within this province; and vice versa.

|  |
| --- |
| **Figure S1: The vaccination data from the Pakistan Population and Health census reported from 2004-2012.** For reporting districts, these were grouped by province with the mean value for the province shown in the dark line. The range of reported immunization rates from the districts in the province are shown in the shaded polygon. Balochistan (Baloc) has the lowest vaccination rates, with Islamabad (ICT), Punjab, and KPK on average have the highest vaccination rates. Although the vaccination has gone down in the eight years of the data in some provinces (such as Balochistan), in general the vaccination rates have remained fairly stable. |

|  |
| --- |
| **Figure S2: The estimated proportion susceptible population per province versus the total travel time estimated from the mobile phone data.** Balochistan (Balo) and Islamabad (ICT) have the lowest amount of incoming travel, although they vary significantly by their proportion susceptible. Punjab has the largest amount of incoming travel, although a low proportion susceptible. Here the size of the point reflects the peak week of reported cases. |

|  |
| --- |
| **Figure S3: The estimated proportion susceptible population per district versus the total travel time estimated from the mobile phone data.** For each district analyzed, we plotted the proportion of the population susceptible against the amount of incoming travel from the mobile phone data. Here the size of the point reflects the peak week of reported cases. |

| 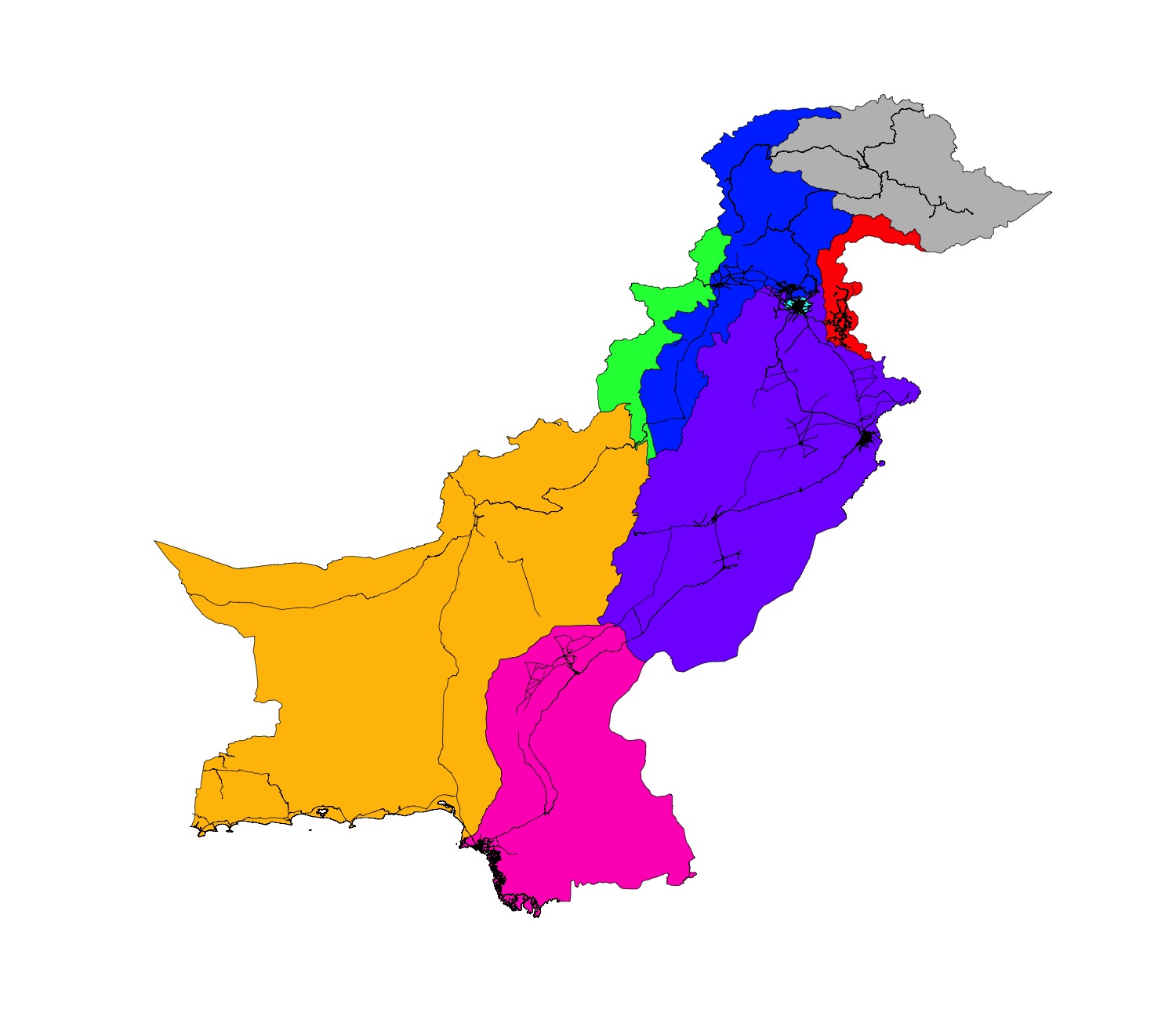 |
| --- |
| **Figure S4: The province map of Pakistan with major national roads in black.** |

*Comparison between outbreaks and alert case data*

|  |
| --- |
| **Figure S5: The age structure of outbreak data.** The outbreak data also listed the age bracket and gender for each outbreak case (aged <5 years, 5+ years, M(male)/F(female)). For an average week, there is a slightly higher percentage of male cases (52% vs 48%). Cases were predominantly in children less than 5, although ~1/3 of cases were in children older than five (M: 67% of cases in children <5 years, F: 64% of cases in children <5 years). |

**Figure S6: Scaling connectivity matrices.** Dashed lines show the summed absolute difference between the median and quartiles of the simulation and the observed for 100 simulations (y axis) plotted against a scalar multiplying the connectivity matrix for the flat connectivity matrix (blue) the gravity model connectivity matrix (black) and the mobile phone connectivity matrix (red). Solid lines show smooth splines fitted to these patterns and used to identify the value of the scalar corresponding to the minima.

**Figure S7: Modulating susceptibility.** The predicted timing of the start of measles outbreaks across districts in Balochistan is higher than observed (an average of 9 biweeks) for gravity and mobile phone based connectivity matrices (Figure 4). If we double, or triple susceptibility in Balochistan relative to that estimated in the main text, we can move the timing of outbreaks in this province closer to those observed, indicating how over-estimates of vaccination coverage can modulate our predictions of timing.

| **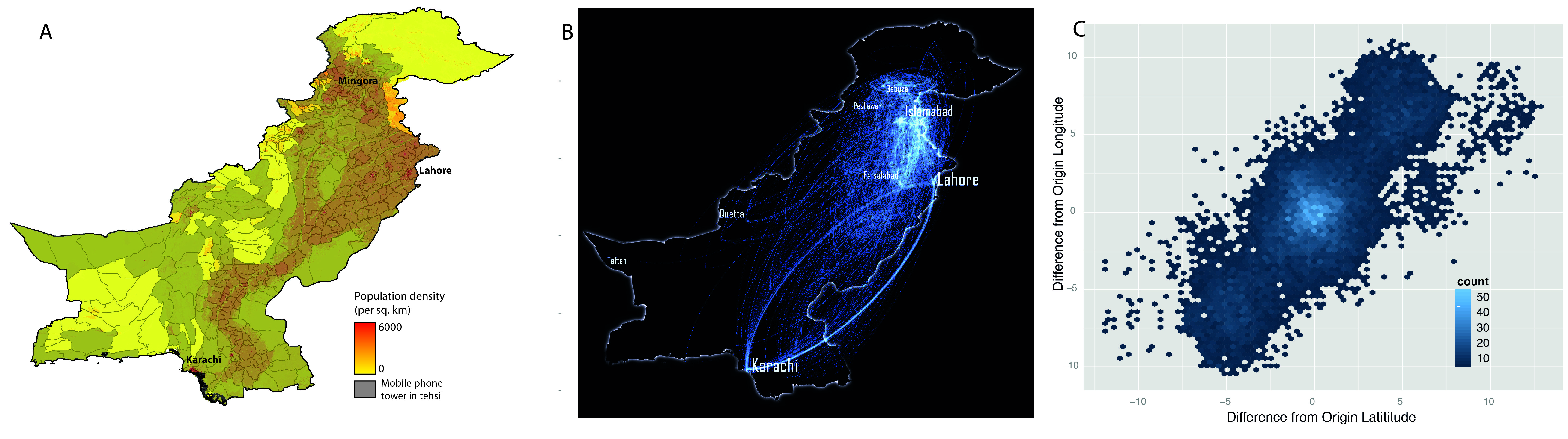** |
| --- |
| **Figure S8: Coverage in Pakistan for mobile phone operator.** For each tehsil, which corresponds to admin level 3 in Pakistan, if the tehsil has a mobile phone tower, from the single operator analyzed in these data, the tehsil is shaded in grey. Population density (red = high, yellow = low) is also shown. All provinces, except Gilgit-Baltistan and Azad Jammu and Kashmir have at least one mobile phone tower. |

| **Table S1: Vaccination coverage values (percentage) for each province (All) from 2004-2012.** These values were obtained from the Pakistan Population and Health Census. For a number of provinces, vaccination coverage estimates were unavailable. | | | | | |
| --- | --- | --- | --- | --- | --- |
| **Province** | **2004** | **2006** | **2008** | **2010** | **2012** |
| Azad Jammu & Kashmir | Not reported | Not reported | Not reported | Not reported | Not reported |
| Balochistan | 62 | 54 | 44 | 58 | 55 |
| Federally Administered Tribal Areas | 77 | 76 | Not reported | Not reported | Not reported |
| Gilgit-Baltistan | Not reported | Not reported | Not reported | Not reported | Not reported |
| Islamabad (ICT) | 89 | 99 | 92 | 83 | 90 |
| Khyber Pakhtunkhwa | Not reported | Not reported | 75 | 78 | 77 |
| Punjab | 89 | 84 | 86 | 86 | 89 |
| Sindh | 73 | 66 | 70 | 77 | 77 |

| **Table S2: The regression results predicting the peak timing of the 2012-2013 outbreak for each district using the proportion of the population susceptible and the amount of incoming travel.** The overall model fit: multiple R-squared = 0.0305, Adjusted R-squared: -0.002, F-statistic = 0.9281 on 2 and 59 DF, p-value = 0.0401. | | | | |  |
| --- | --- | --- | --- | --- | --- |
| Variable | Estimated | Std. Error | t-value | Pr(>\|t\|) |  |
| Intercept | 74.88 | 29.886 | 2.506 | 0.015 * |  |
| Proportion Susceptible | 173.8 | 1.586 | 0.658 | 0.513 |  |
| Amt of Incoming Travel (Log) | 1.044 | 148.150 | 1.173 | 0.245 |  |
| Signif. codes: 0 ‘***’ 0.001 ‘**’ 0.01 ‘*’ 0.05 ‘.’ 0.1 ‘ ’ 1 | | | | |  |
|  | | | | | |
